# Editorial: Bringing together the worlds of photosynthesis and photovoltaics: mechanisms, methods, and applications

**DOI:** 10.3389/fpls.2023.1321591

**Published:** 2023-10-24

**Authors:** Suleyman I. Allakhverdiev, Jean Manca, Alfred Holzwarth, Janne Halme, Raoul N. Frese, Roland Valcke

**Affiliations:** ^1^ Controlled Photobiosynthesis Laboratory, Institute of Plant Physiology, Russian Academy of Sciences, Moscow, Russia; ^2^ X-LAB, University of Hasselt, Diepenbeek, Belgium; ^3^ Max Planck Institute for Chemical Energy Conversion, Mülheim, Germany; ^4^ Department of Applied Physics, Aalto University School of Science, Espoo, Finland; ^5^ Biophysics of Photosynthesis Research Group, Vrije Universiteit Amsterdam, Amsterdam, Netherlands; ^6^ Molecular and Physical Plant Physiology, University of Hasselt, Diepenbeek, Belgium

**Keywords:** charge-transfer state, biophotovoltaic, agriphotovoltaic, photosynthesis, crystal structure, in silico light simulation, photoprotection, phenomenological fluxes

Solar energy is considered as the driving force not only for photosynthesis but also for photovoltaic cells. Both systems are structurally completely different but share common mechanisms. The basic units of the photosynthetic machinery are the pigment-protein-complexes, known as photosystem I (PSI) and II (PSII) and the light-harvesting complex (LHCP). Arranged in highly organized manner and embedded in thylakoid membranes, sunlight will be absorbed and converted in chemical energy (as ATP) and reducing power (as NADPH) driven by electron transport in the so called light reactions. These two compounds will then be used to reduce CO_2_ to simple sugars in the Calvin-Benson-Bassham cycle which forms the basis for the synthesis of more complex organic molecules. Together with these metabolic reactions, an important waste product, oxygen, is formed by the oxygen evolving complex (OEC), which is part of the photosystem II complex (Long et al., 2022).

So, photosynthesis (PHS) converts solar energy into chemical energy, yielding a variety of products, including essential building blocks, biofuels, and biomass (Nikolaidis, 2023; Voloshin et al., 2023a; Voloshin et al., 2023b). In contrast, PVs transform sunlight into electricity, which can be stored and utilized for various applications. Recent years have witnessed significant advancements in our understanding of the fundamental processes involved in capturing light energy in both natural PHS and PV (El-Khouly et al., 2017). While these domains share common aspects such as light absorption, charge separation, and charge transport mechanisms, their collaboration has remained somewhat limited. However, bridging these two realms holds the promise of mutual benefits, enhancing our fundamental understanding, refining characterization techniques, and expanding the horizons of their applications (Martínez-Huitle et al., 2023; Sadvakasova et al., 2023; Voloshin et al., 2023a; Voloshin et al., 2023b). A cross-disciplinary exploration into light-harvesting structures and their interaction with quantum phenomena, such as excitonic states and quantum coherence, is expected to yield novel insights. This deeper comprehension of natural photosynthetic complexes may inspire the development of artificial photosynthetic systems (APSs) and innovative solar energy conversion technologies (Balouch et al., 2023; Voloshin et al., 2023b). A wide range of bioinspired concepts and applications is emerging, encompassing endeavors from light-induced water splitting to plant microbial fuel cells (PMFCs) and hybrid systems that integrate PHS and PV. These hybrids hold great potential, especially in agriphotovoltaic (APV) concepts, where solar cells coexist with plants, and transparent solar cells are positioned in front of or above the vegetation (Osorio de la Rosa et al., 2019).

The objective of this Research Topic is to disseminate original research papers, comprehensive reviews, and expert opinions that facilitate the convergence of the realms of photosynthesis and PV, fostering cross-disciplinary dialogue between these two research communities. Furthermore, this Research Topic delves into the contemporary challenges, lingering inquiries, and future trajectories within the domains of PHS and PV, offering insights into ongoing issues in these fields. While the current Research Topic is relatively concise, it boasts two comprehensive reviews, five original research articles, and one thought-provoking opinion piece.

Additionally, this Research Topic showcases the contributions of 47 authors hailing from 11 diverse countries: Austria – 1, Bangladesh – 1, Belgium – 6, China – 16, Czech Republic – 1, Denmark – 1, Greece – 1, India – 11, Japan – 4, Netherlands – 1, and the United Kingdom – 2. From the list of corresponding authors, it is noteworthy that three articles originate from China, two from Japan, one from India, one from Belgium, and one from South Korea.

The opinion article by Zhu et al. aptly highlights the historical significance of PHS discoveries while underlining its current relevance and future potential. The article’s emphasis on PHS as a pivotal solution for contemporary challenges, including sustainable agriculture and ecological stability, is commendable. The call to develop more efficient photosynthetic systems resonates with the growing need for sustainable resource utilization. It underscores the profound impact of PHS research in addressing pressing global issues, making it an exciting and vital field for future scientific endeavors.

Furthermore, in this Research Topic, a study investigating the characteristics of biofilms aimed at modern agricultural practices has also been published. For instance, Liu et al. have introduced an innovative approach to address uneven crop growth in Chinese Solar Greenhouses (CSG) by utilizing reflective films. They developed a sophisticated 3D greenhouse model to simulate the effects of these films on the microlight climate and crop growth, resulting in precise predictions of solar radiation intensity over time and different positions. The findings suggest that customized configurations of reflective films can significantly enhance light distribution within the tomato canopy, offering a promising strategy for improving CSG productivity and potentially benefiting other agricultural settings.

Additionally, in this Research Topic, three distinct studies focused on PHS and its light-harvesting structures are presented, collectively shedding light on essential aspects. First, Shanker et al.’s review delivers a comprehensive and well-structured examination of the impact of Heat and Water Deficit Stress on PHS in pearl millet plants. The authors exhibit a profound command of the subject matter and provide clear insights into the effects of these stressors on the electron transport system in Photosystem II (PSII). Their differentiation between the effects of heat and water stress, as well as their combined influence, represents a valuable addition to the field. Overall, it stands as an invaluable resource for researchers in the realm of plant physiology.

Second, the original research conducted by Sugo and Ishikita illuminates the molecular basis of QA·– stabilization in PSII, with a particular emphasis on the pivotal role of proton mediation. The discovery of a low-barrier hydrogen bond between D2-His214 and QA·–, coupled with insights into bicarbonate protonation and decomposition, enriches our understanding of photoprotection mechanisms. These findings carry implications for optimizing photosynthetic processes, potentially enhancing efficiency, and devising photoprotective strategies. In essence, this study significantly advances our comprehension of PSII functionality and its potential applications across agriculture and renewable energy.

Last, Saito et al.’s research delves into the differences in hydrogen bond distances within the Mn_4_CaO_5_ cluster in PSII by employing a QM/MM approach. It suggests that variations in oxidation states between monomer units A and B may underlie these disparities. These findings offer valuable insights into the complex structural dynamics of PSII, furthering our understanding of how this vital photosynthetic complex operates and responds to different states. Such insights may have far-reaching implications for harnessing PHS for various applications.

In this Research Topic, three illuminating articles dedicated to PV energy are presented: one research article and two review papers. The study by Moon and Ku delves into the influence of APV systems on broccoli production. It underscores the significance of visual attributes, metabolite profiles, and yield while considering additional shading treatments and seasonal variations. The results unveiled intriguing insights: glucosinolate content’s susceptibility to seasonal and cultivar variations, shading’s positive impact on visual qualities and chlorophyll content, as well as reduced anthocyanin levels and elevated aspartic acid content with shading. These findings underscore the potential of APV systems to enhance crop aesthetics and shed light on metabolic shifts in broccoli under diverse conditions.

Meanwhile, Ge-Zhang et al.’s concise review delves into the burgeoning field of biophotovoltaics (BPV). It succinctly outlines BPV’s advantages as a clean energy technology, highlighting its potential to address environmental concerns associated with conventional PV. The review delves into the biological materials utilized in BPV, strategies to surmount low photocurrent challenges, and identifies limitations, enriching our grasp of this innovative approach. In essence, this mini-review serves as an informative resource, accentuating BPV’s significance in sustainable energy research.

Last, a review provided by Hustings et al. draws attention to the analogous charge-transfer mechanisms in natural PHS and synthetic organic PV. Despite disparate research communities and terminology, similarities emerge in how light-induced charge-transfer complexes function, spanning from light absorption to charge separation. The review aspires to bridge these disciplinary divides by suggesting standardized terminology and promoting interdisciplinary cooperation. This approach aims to kindle the development of more efficient solar cells and deepen our comprehension of charge-transfer processes in both natural and artificial light-harvesting systems, ultimately benefiting both domains.

In summary, this Research Topic explores the synergy between PHS and PV, showcasing advancements, potential applications, and innovative approaches. It highlights the importance of cross-disciplinary collaboration and offers insights into optimizing solar energy capture and utilization. We aspire that the readers will derive value from the research presented here, igniting their innovative inquiry, fostering fresh revelations, and deepening their comprehension of the intricate mechanisms of photosynthesis. It is our intent that this knowledge will equip them with effective approaches to tackle global challenges concerning food security, sustainable energy, and climate change.

## Author contributions

RV: Writing – review & editing. JM: Writing – review & editing. AH: Writing – review & editing. JH: Writing – review & editing. RF: Writing – review & editing. SA: Writing – original draft.

## References

[B1] Balouch H. Zayadan B. K. Sadvakasova A. K. Kossalbayev B. D. Bolatkhan K. Gencer D.. (2023). Prospecting the biofuel potential of new microalgae isolates. Int. J. Hydrogen Energy 48, 19060–19073. doi: 10.1016/j.ijhydene.2023.02.028

[B2] El-Khouly M. E. El-Mohsnawy E. Fukuzumi S. (2017). Solar energy conversion: From natural to artificial photosynthesis. J. Photochem. Photobiol. C: Photochem. Rev. 31, 36–83. doi: 10.1016/j.jphotochemrev.2017.02.001

[B3] Long S. P. Taylor S. H. Burgess S. J. Carmo-Silva E. Lawson T. De Souiza A. P.. (2022). Into the shadows and back into sunlight: photosynthesis in fluctuating light. Annu. Rev. Plant Biol. 73, 617–648. doi: 10.1146/annurev-arplant-070221-024745 35595290

[B4] Martínez-Huitle C. A. Rodrigo M. A. Sirés I. Scialdone O. (2023). A critical review on latest innovations and future challenges of electrochemical technology for the abatement of organics in water. Appl. Catalysis B: Environ. 328, 122430. doi: 10.1016/j.apcatb.2023.122430

[B5] Nikolaidis P. (2023). Solar energy harnessing technologies towards de-carbonization: A systematic review of processes and systems. Energies 16, 6153. doi: 10.3390/en16176153

[B6] Osorio de la Rosa E. Vázquez Castillo J. Carmona Campos M. Barbosa Pool G. R. Becerra Nuñez G. Castillo Atoche A.. (2019). Plant microbial fuel cells–based energy harvester system for self-powered IoT applications. Sensors (Basel) 19, 1378. doi: 10.3390/s19061378 30897710 PMC6470559

[B7] Sadvakasova A. K. Kossalbayev B. D. Bauenova M. O. Balouch H. Leong Y. K. Zayadan B. K.. (2023). Microalgae as a key tool in achieving carbon neutrality for bioproduct production. Algal Res. 72, 103096. doi: 10.1016/j.algal.2023.103096

[B8] Voloshin R. A. Bozieva A. M. Bruce B. D. Allakhverdiev S. I. (2023a). Photosynthetic microbial fuel cells: practical applications of electron transfer chains. Russ. Chem. Rev. 92 (5), RCR5073. doi: 10.57634/RCR5073

[B9] Voloshin R. A. Lokteva E. S. Allakhverdiev S. I. (2023b). Photosystem I in the biohybrid electrodes. Curr. Opin. Green Sustain. Chem. 41, 100816. doi: 10.1016/j.cogsc.2023.100816

